# Maternal and child patterns of Medicaid retention: a prospective cohort study

**DOI:** 10.1186/s12887-018-1242-4

**Published:** 2018-08-21

**Authors:** Susmita Pati, Rose Calixte, Angie Wong, Jiayu Huang, Zeinab Baba, Xianqun Luan, Avital Cnaan

**Affiliations:** 1 Division of Primary Care Pediatrics, State University of New York at Stony Brook, 100 Nicolls Rd, Stony Brook, NY 11794 USA; 2 Pediatric Generalist Research Group, The Children’s Hospital of Philadelphia, 3401 Civic Center Blvd, Philadelphia, PA 19104 USA; 3 Healthcare Analytics Unit, The Children’s Hospital of Philadelphia, 3401 Civic Center Blvd, Philadelphia, PA 19104 USA; 4 School of Medicine and Health Sciences, The George Washington University, 2121 I St NW, Washington, DC 20052 USA; 5 Center for Clinical and Translational Science, Children’s National Medical Center, 111 Michigan Ave NW, Washington, DC 20010 USA

**Keywords:** Medicaid retention, Health insurance, Children

## Abstract

**Background:**

We sought to determine whether maternal Medicaid retention influences child Medicaid retention because caregivers play a critical role in assuring children’s health access.

**Methods:**

We conducted a longitudinal prospective cohort study of a convenience sample of 604 Medicaid-eligible mother-child dyads followed from the infant’s birth through 24 months of age with parent surveys. Individual enrollment status was abstracted from administrative Medicaid eligibility files. Generalized estimating equations quantified the effect of maternal Medicaid enrollment status on child Medicaid retention, adjusting for relevant covariates. Because varying lengths of gaps may have different effects on child health outcomes, Medicaid enrollment status was further categorized by length of gap: any gap, > 14-days, and > 60-days.

**Results:**

This cohort consists primarily of African-American (94%), unmarried mothers (88%), with a mean age of 23.2 years. In multivariable analysis, children whose mothers experienced any gaps in coverage had 12.6 times greater odds of experiencing gaps when compared to children whose mothers were continuously enrolled. Use of varying thresholds to define coverage gaps resulted in similar odds ratios (> 14-day gap = 11.8, > 60-day gap = 16.8). Cash assistance receipt and maternal knowledge of differences between Temporary Assistance to Needy Families and Medicaid eligibility criteria demonstrated strong protective effects against child Medicaid disenrollment.

**Conclusions:**

Medicaid disenrollment remains a significant policy problem and maternal Medicaid retention patterns show strong effects on child Medicaid retention. Policymakers need to invest in effective outreach strategies, including family-friendly application processes, to reduce enrollment barriers so that all eligible families can take advantage of these coverage opportunities.

## Background

Children with health insurance coverage gaps are less likely than those with continuous coverage to have access to a regular source of care for routine preventive needs (e.g., well-child care visits, developmental screening, immunizations) [1, 2]. This phenomenon contributes to poor health outcomes [1, 2]. In fact, children with brief health insurance coverage gaps have comparable health outcomes to children who are continuously uninsured [3, 4]. In recent years, many states have simplified enrollment and renewal procedures for public insurance programs to reduce the number of eligible children losing coverage for procedural reasons [5, 6]. However, coverage gaps affected as many as 33–40% of children transitioning from Medicaid-based public insurance plans to separate Children’s Health Insurance Program public insurance plans [7].

Individual characteristics and policy-level factors are known to influence child Medicaid retention. Our work in this study is theoretically grounded in Anderson and Aday’s widely used framework for studying access to care that highlights the interaction between the organization of health care services and individual characteristics that affect access to care [8]. For instance, Hispanic children and older children are disproportionately more likely than their peers to experience coverage gaps [4, 9–11]. At the policy level, the 1997 passage of welfare reform that separated cash assistance (i.e. Temporary Assistance to Needy Families [TANF]) and Medicaid eligibility resulted in significant confusion about eligibility and application processes that, in turn, resulted in significant drops in enrollment in both programs [12, 13]. In addition, one recent study revealed that with only one exception all state Medicaid renewal applications in 2008 were written at the fifth grade level or higher, suggesting that poor caregiver literacy may adversely affect child Medicaid retention [14]. Several studies have associated parental health insurance status with that of their children, but did not include individual-level information about parental health literacy or TANF eligibility [15–17]. Though children rely on caregivers to initiate enrollment and complete renewals, the direct longitudinal influence of maternal Medicaid enrollment status on child Medicaid retention has not been well quantified in population-based studies.

The primary hypothesis of this study was that maternal Medicaid disenrollment increases the likelihood of child Medicaid disenrollment. We also explored various thresholds for defining coverage gaps and quantified the time to the child’s first disenrollment to better understand this relationship. Our secondary goal was to advance our understanding of the influence of other plausible factors on child Medicaid retention that have not been fully explored to date. These factors include maternal health literacy, cash assistance receipt, and maternal knowledge about the separation of eligibility determinations for TANF and Medicaid. In this study, we focused on the association between maternal and child disenrollment, for any reason, because this issue is critical from the perspectives of patients and providers.

## Methods

### Study design, study population and data sources

We performed a prospective cohort study of mother-infant dyads enrolled in the Health Insurance Improvement Project (HIP). The overarching aim of the HIP study was to identify individual characteristics and policy factors that influence child Medicaid retention. This study was approved by and carried out in accordance with guidelines from the Institutional Review Boards at the University of Pennsylvania, The Children’s Hospital of Philadelphia, and Stony Brook University. Between June 2005 and August 2006, study subjects who were enrolled or eligible for Medicaid as indicated in the hospital medical record were recruited as a convenience sample from the post-partum wards of a large urban hospital shortly after the infant’s birth. As previously published, we enrolled 744 of the 1395 eligible mother-child dyads (Figure 2 in Appendix 1) [18]. If multiple children (e.g., twins) were born to the same mother, only one child was chosen randomly to be included in the study. Upon enrollment, mothers completed a baseline survey, which included socio-demographic information and the Short-Test of Functional Health Literacy in Adults (S-TOFHLA) [19, 20]. Subsequently, a computer-assisted survey instrument was administered via telephone every 6 months through age 24 months by trained staff to collect data about additional covariates. We obtained informed consent from all subjects in accordance with guidelines from the Institutional Review Boards at the aforementioned institutions.

### Measures

Our primary predictor of interest was maternal Medicaid disenrollment and the primary outcome of interest was child Medicaid disenrollment. We linked administrative Medicaid eligibility data (including category of eligibility as well as enrollment and termination dates) for mothers and children using individual identifiers collected at enrollment. We defined the start of each subject’s observation period as the child’s date of birth and the end as 6 months after the last follow-up survey administered. We assumed all subjects had Medicaid coverage from birth through the end of the observation period except for those with a Medicaid termination date in the eligibility data occurring earlier than the end of the observation period. We censored observations on subjects who reported moving out of state, entering foster care or adoption services, at the time point of the event. Of the 744 enrolled dyads, we successfully linked 604 (81.2%) to administrative Medicaid eligibility files and this group comprised the analytic study sample. Notably, matched mother-child dyads were more likely to be U.S. born than unmatched subjects (Table 6 in Appendix 2).

We defined disenrollment as any period without Medicaid coverage at any time during the observation period. Notably, there were 48 infants who were not enrolled in Medicaid at birth (mean number of days to first enrollment after birth: 84.7, standard deviation 139.7). For these infants, we considered this gap between birth and first enrollment their first disenrollment. We used three different thresholds to define uninsured periods for the child: any gap (i.e. any period without coverage), > 14 days gap, and > 60 days gap. We selected the 14 day threshold because the American Academy of Pediatrics recommends that newborns have three health supervision visits in the first 2 weeks of life [21] and we generalized this threshold to all ages because any gap in coverage for young children may adversely affect health care access and, in turn, outcomes. We selected the 60 day threshold because Medicaid agencies can take up to 45 days to process an application [22]. We applied the ‘any gap’ definition to maternal Medicaid coverage data in order to keep the definition of maternal disenrollment consistent across models. We did not classify switches from one eligibility category to another while maintaining coverage as a disenrollment event. For purposes of determining enrollment trends, we recorded any change in enrollment status (e.g., disenrollment, re-enrollment) in either the mother or the child as a unique period. Notably, each subject could have multiple enrollment periods (e.g., enrolled April 2006–September 2006, disenrolled October 2006–November 2006, re-enrolled December 2006–March 2007, etc.). For each period, we classified Medicaid eligibility categories as cash-assistance (i.e. TANF or Supplemental Security Income) related or not.

### Covariates

We collected covariates known to influence Medicaid and/or public program participation [10, 12, 23–26] in the following ways. We collected socio-demographic information using items adapted from the National Health Interview Survey administered at birth in-person and then every 6 months via telephone for the remainder of the study period [27]; maternal health literacy (using S-TOFHLA) [19, 20] and maternal knowledge that Medicaid and TANF have different eligibility criteria were collected in person at enrollment. We assessed maternal instrumental and relational social support using scores from the Maternal Social Support Index (MSSI) that was administered at 12, 18, 24 months after enrollment; a higher score indicates greater social support [28].

### Statistical analyses

The main goal of our analyses was to assess whether maternal Medicaid disenrollment was significantly associated with child Medicaid disenrollment status after adjusting for relevant covariates. We included all covariates except maternal age (continuous) as categorical variables. We treated maternal health literacy and maternal knowledge that Medicaid and TANF have different eligibility as fixed covariates. Maternal health status, employment status, social support, household income, and housing situation changed over time and were included as time varying covariates. We treated all other factors as fixed covariates using values obtained at the 6-month survey consistent with the observed patterns in the data. When child disenrollment occurred, we used the most recent covariate and mother disenrollment data.

We used generalized estimating equations (GEE) to determine how well child Medicaid enrollment status could be explained by maternal Medicaid enrollment status and the covariates. In the GEE, the child was the cluster and the cluster had as many observations as there were enrollment or disenrollment periods. Thus, a child who was continuously enrolled in Medicaid had one observation in the cluster. If a child disenrolled once and never reenrolled, that child had two observations in the cluster. We calculated odds ratios (ORs) for child disenrollment based on the GEE models to assess the impact of each covariate. We performed sensitivity analyses to test whether the definition of gap (any, > 14 days, > 60 days) affects maternal and child Medicaid enrollment status. We used a best subsets approach to create and choose the best fitting models in order to obtain the most parsimonious and best fitting model that explains child Medicaid enrollment status [29]. We checked the final models to confirm no collinearity problem was present. We examined model fit using the Quasi-likelihood under Independence Model Criterion (QIC) [30, 31].

We next used the Cox proportional hazards model to determine the response of ‘time to child’s first Medicaid disenrollment’ to maternal Medicaid enrollment status and covariates. Here, we used ‘any gap’ to define uninsured periods for both child and maternal Medicaid coverage. All changes in time-varying covariates were recorded. We used the most recent covariates and mother disenrollment data when child’s disenrollment occurred. We censored a child continuously enrolled in Medicaid during the study period. A child not enrolled in Medicaid on the date of birth had time-to-disenrollment of 0 days. We checked the proportional hazards assumption for each covariate. We calculated hazard ratios for time to child disenrollment based on the Cox proportional hazards model to assess the impact of predictors. We used a best subsets approach to create and choose the best fitting models. There were no collinearity problems in the final models. We examined model fit by Akaike information criterion (AIC).

### Missing data

The fraction of missing survey data ranged from 0.17 to 16.06% per item, with variables for maternal employment and knowledge that Medicaid and TANF eligibility criteria differ having more than 10% missing. We performed multiple imputation for missing data using the method of chained equations [32]. To avoid potential bias and potential reduction in statistical power from using only complete observations. All reported results, including standard errors, are from completed datasets using the imputation procedures.

A Type I error level of 0.05 was used for all analyses, and all significance tests were two-sided. SAS 9.3® was used for analyses.

## Results

The analytic sample cohort consists mostly of young, African-American mothers with more than one child who were not married (Table 1). The majority completed high school, had adequate health literacy, and knew that eligibility criteria for TANF and Medicaid differ. More than half of mothers were unemployed or students, did not live in their own housing, and had household incomes of <$1000 per month. Among mothers who completed the MSSI (*n* = 478), most mothers reported to having medium to high social support.

**Table 1:** Population characteristics at child’s birth and association with child Medicaid disenrollment

Characteristics	N (%)	Any gap			> 14 day gap			> 60 day gap		
		Children with no gap N(%)	Children with gap N(%)	*P*-value^a^	Children with no gap N(%)	Children with gap N(%)	*P*-value^a^	Children with no gap N(%)	Children with gap N(%)	*P*-value^a^
Total sample	604	450(75)	154(25)		469(78)	135(22)		502(83)	102(17)	
Child gender										
Male	308(51)	224(50)	84(55)	0.30	236(50)	72(53)	0.53	254(51)	54(53)	0.66
Female	296(49)	226(50)	70(45)		233(50)	63(47)		248(49)	48(47)	
Maternal age										
Under 18	54(9)	39(9)	15(10)	0.92	40(9)	14(10)	0.74	42(8)	12(12)	0.48
18–24	349(58)	261(58)	88(57)		274(58)	75(56)		294(59)	55(54)	
25 and over	201(33)	150(33)	51(33)		155(33)	46(34)		166(33)	35(34)	
Maternal race										
African American	569(94)	427(95)	142(92)	0.21	444(95)	125(93)	0.36	475(95)	94(92)	0.33
Other^b^	35(6)	23(5)	12(8)		25(5)	10(7)		27(5)	8(8)	
Maternal education										
Less than High School	198(33)	153(34)	45(29)	0.51	159(34)	39(29)	0.54	168(33)	30(29)	0.69
High School	147(24)	109(24)	38(25)		113(24)	34(25)		122(24)	25(25)	
More than High School	259(43)	188(42)	71(46)		197(42)	62(46)		212(42)	47(46)	
Maternal health literacy*										
Inadequate	56(9)	38(8)	19(12)	<.0001	41(9)	16(12)	0.0021	46(9)	11(11)	0.05
Marginal	80(13)	58(13)	21(14)		61(13)	19(14)		69(14)	11(11)	
Adequate	467(77)	353(78)	115(75)		367(78)	100(74)		387(77)	80(78)	
Other children in household										
None	228(38)	158(35)	70(45)	0.073	169(36)	59(44)	0.26	181(36)	47(46)	0.062
One	157(26)	122(27)	35(23)		126(27)	31(23)		139(28)	18(18)	
Two or more	219(36)	170(38)	49(32)		174(37)	45(33)		182(36)	37(36)	
Prenatal Care, self-reported*										
All/Most of the time	553(92)	411(91)	142(92)	0.28	428(91)	125(93)	0.11	458(91)	95(93)	0.04
Some/None of the time	51(9)	39(9)	12(8)		41(9)	10(7)		44(9)	7(7)	
Maternal self-reported health										
Score < 80 (poor health)	224(37)	163(36)	61(40)	0.45	169(36)	55(41)	0.32	181(36)	43(42)	0.24
Score ≥ 80 (good health)	380(63)	287(64)	93(60)		300(64)	80(60)		321(64)	59(58)	
Maternal knowledge that TANF and Medicaid eligibility criteria are different										
Yes	492(81)	377(84)	115(75)	<.0001	387(83)	105(78)	<.0001	412(82)	80(78)	0.006
No	112(19)	73(16)	39(25)		82(17)	30(22)		90(18)	22(22)	
Travel time to Medicaid office*										
< 30 min	410(68)	305(68)	105(68)	0.93	320(68)	90(67)	0.73	341(68)	69(68)	0.96
≥ 30 min	194(32)	145(32)	49(32)		149(32)	45(33)		161(32)	33(32)	
Household income*										
< $1000/month	410(68)	312(69)	98(64)	<.0001	325(69)	85(63)	<.0001	350(70)	60(59)	<.0001
$1000 or more/month	194(32)	138(31)	56(36)		145(31)	50(37)		152(30)	42(41)	
Marital status										
Single/Divorced/Widowed	533(88)	404(90)	129(84)	0.045	422(90)	111(82)	0.013	452(90)	81(79)	0.002
Married	71(12)	46(10)	25(16)		47(10)	24(18)		50(10)	21(21)	
Maternal employment status*										
Student	152(25)	118(26)	34(22)	<.0001	123(26)	29(21)	<.0001	127(25)	26(25)	0.0002
Full Time	245(41)	172(38)	72(47)		181(39)	63(47)		198(39)	47(46)	
Unemployed	207(34)	160(36)	48(31)		164(35)	43(32)		177(35)	30(29)	
Family housing situation										
Lives in own housing	243(40)	234(52)	61(40)	0.890	245(52)	51(38)	0.40	262(52)	38(37)	0.30
Rents or lives with relative	361(60)	216(48)	93(60)		224(48)	84(62)		240(48)	64(63)	
Maternal social support*										
Low	190(32)	134(30)	56(36)	<.0001	143(30)	47(35)	<.0001	157(31)	33(32)	<.0001
Medium	135(22)	98(22)	37(24)		102(22)	33(24)		108(22)	27(26)	
High	153(25)	102(23)	51(33)		106(23)	47(35)		113(23)	40(39)	
Not collected	126(21)	116(26)	10(7)		118(25)	8(6)		124(25)	2(2)	

Notes: Maternal health literacy was assessed using the S-TOFHLA and categorized as inadequate, marginal, or adequate per published technical guidance (Nurss JR, Parker R, Willams M, Baker D. *TOFHLA* Test of Functional Health Literacy in Adults. Second ed. Snow Camp, NC: Peppercorn Books & Press; 2001). Maternal self-reported health was assessed using the SF-36® (Ware JE, Jr., Sherbourne CD. The MOS 36-item short-form health survey (SF-36). I. Conceptual framework and item selection. *Med. Care*. Jun 1992;30(6):473–483). Maternal instrumental and relational social support was assessed using the Maternal Social Support Index and categorized low, medium, or high using tertiles per published technical guidance (Pascoe JM, Ialongo NS, Horn WF, Reinhart MA, Perradatto D. The reliability and validity of the maternal social support index. *Fam. Med*. Jul-Aug 1988;20(4):271–27)

^a^*p*-value is for the χ2 test of association

^b^Includes Hispanic, Asian and “Ethnically-Challenged” (as self-reported)

*Results from 10 imputed datasets. Percentages may not total 100 due to rounding

Table 1 also shows that the number and proportion of children with gaps in Medicaid coverage changed as the threshold for defining coverage gaps varied. One-quarter (*n* = 154) of children experienced at least one gap of any length of time in coverage during the first two years of life. Among those with any gap, 135 (22% of total) children were disenrolled for > 14 days and 102 (17% of total) children were disenrolled for > 60 days. Generally, the characteristics of children with gaps were similar regardless of the threshold used to define coverage gaps. Continuously enrolled children were more likely than children with any gap in coverage—regardless of the threshold used to define gaps-- to have single, unemployed mothers with adequate health literacy, who know that Medicaid and TANF eligibility criteria differ, have low household income and have low social support.

**Fig. 1:**
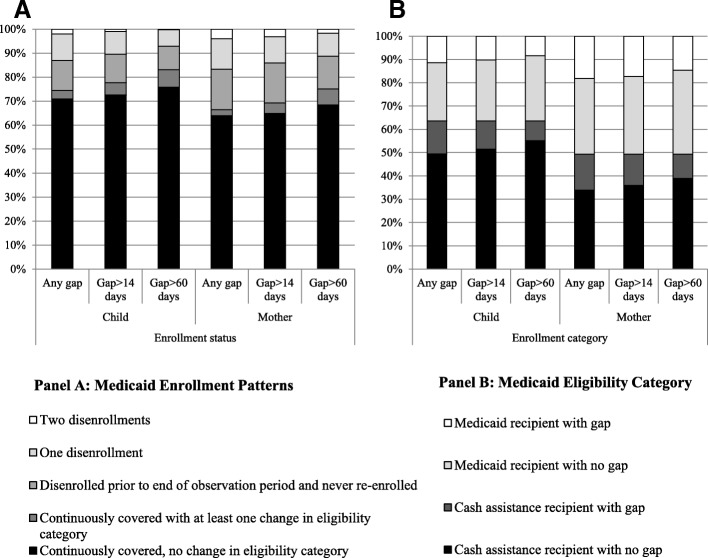
Medicaid enrollment patterns and eligibility category for mothers and children, by gap definition. Panel **a** Medicaid Enrollment Patterns. Panel **b** Medicaid Eligibility Category Notes**:** Disenrollment was defined as any period without Medicaid coverage at any time during the observation period. Results from 10 imputed datasets.

**Fig. 2:**
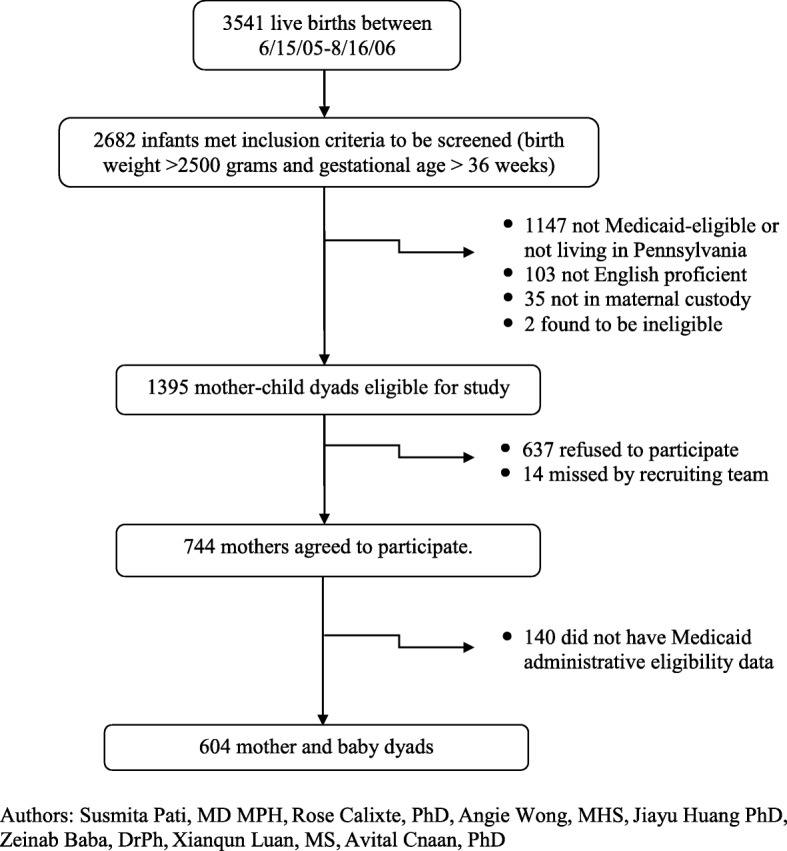
Study enrollment protocol

Regardless of the length of gap, the distribution of coverage patterns for mothers and children were similar with a greater proportion of mothers experiencing coverage difficulties than children (Fig. 1 Medicaid enrollment patterns and eligibility category for mothers and children, by gap definition, Panel a Medicaid enrollment patterns). Most children and mothers were continuously covered with no change in eligibility category across different gap definitions. When exploring the distribution of Medicaid eligibility categories, specifically cash assistance recipients vs. Medicaid only, we observed that a greater proportion of children than mothers were cash assistance recipients (Fig. 1 Medicaid enrollment patterns and eligibility category for mothers and children, by gap definition, Panel b Medicaid eligibility category). Comparing mothers and children who received cash assistance, a consistently greater proportion of mothers than children experienced gaps of any length. At the same time, more mothers who were Medicaid-only recipients had coverage gaps than mothers who received cash assistance.

Maternal disenrollment and mother-child cash assistance receipt were strongly and significantly associated with child disenrollment, regardless of the threshold used to define gaps in coverage for children (Table 2**)**. Specifically, maternal disenrollment (defined as any gap in Medicaid coverage based on sensitivity analyses performed) was associated with a more than 10 times increased odds of child disenrollment at all three thresholds for defining gaps. Mother-child cash assistance receipt and child cash assistance receipt had similarly strong protective effects against child disenrollment at all gap levels. Children of married mothers were more likely than others to have gaps > 14 days or > 60 days. Maternal lack of knowledge that Medicaid and TANF have different eligibility criteria was associated with increased odds of child having any gap, but not with gaps > 14 or > 60 days. Similarly, children whose families were renting or living with friends/relatives were more likely than those living in their own housing to have any gap, but not gaps > 14 or > 60 days. Notably, maternal health literacy was not significantly associated with child disenrollment.

**Table 2:** Odds ratios for predictors of child Medicaid disenrollment based on single predictor variable GEE models

Variable	Any gap		> 14 day gap		> 60 day gap	
	Odds Ratio (95% CI)	*P*-value	Odds Ratio (95% CI)	*P*-value	Odds Ratio (95% CI)	*P*-value
Maternal disenrollment						
Under Medicaid coverage	Ref.	–	Ref.	–	Ref.	–
Disenrolled from Medicaid (any gap)	**11.97 (8.09, 17.72)**	**< 0.001**	**10.81(7.09, 16.48)**	**< 0.001**	**15.76(9.71, 25.58)**	**< 0.001**
Child cash assistance status						
No	Ref.	–	Ref.	–	Ref.	–
Yes	**0.29 (0.20, 0.41)**	**< 0.001**	**0.33(0.23, 0.48)**	**< 0.001**	**0.29(0.19, 0.45)**	**< 0.001**
Mother cash assistance status						
No	Ref.	–	Ref.	–	Ref.	–
Yes	0.77(0.56, 1.06)	0.11	0.87(0.62, 1.23)	0.44	0.84(0.56, 1.24)	0.37
Combined cash assistance status						
Neither had cash assistance	Ref.	–	Ref.	–	Ref.	–
Only mother had cash assistance	1.47(0.92, 2.34)	0.10	**1.71(1.03, 2.82)**	**0.036**	1.59(0.92, 2.76)	0.099
Only child had cash assistance	**0.30(0.18, 0.52)**	**< 0.001**	**0.37(0.21, 0.65)**	**< 0.001**	**0.30(0.15, 0.58)**	**< 0.001**
Both had cash assistance	**0.32(0.21, 0.49)**	**< 0.001**	**0.39(0.25, 0.60)**	**< 0.001**	**0.35(0.21, 0.58)**	**< 0.001**
Maternal age						
	0.99(0.95, 1.02)	0.41	0.99(0.96, 1.03)	0.66	0.99(0.95, 1.03)	0.61
Maternal race						
African American	Ref.	–	Ref.	–	Ref.	–
Other	1.09(0.75, 1.57)	0.65	1.17(0.77, 1.77)	0.46	1.18(0.74, 1.89)	0.48
Marital status						
Single/widowed/divorced	Ref.	–	Ref.	–	Ref.	–
Married	1.56(0.90, 2.70)	0.11	**1.88(1.07, 3.30)**	**0.029**	**2.23(1.22, 4.07)**	**0.0090**
Maternal health literacy^*^						
Inadequate	Ref.	–	Ref.	–	Ref.	–
Marginal	0.71(0.38, 1.33)	0.29	0.79(0.40, 1.60)	0.52	0.74(0.31, 1.73)	0.48
Adequate	0.79(0.49, 1.29)	0.35	0.76(0.43, 1.34)	0.34	0.87(0.44, 1.71)	0.66
Maternal education						
Less than High School	Ref.	–	Ref.	–	Ref.	–
High School	1.08(0.71, 1.66)	0.70	1.11(0.69, 1.76)	0.66	1.01(0.59, 1.71)	0.97
More than High School	1.10(0.76, 1.59)	0.60	1.08(0.72, 1.62)	0.72	0.96(0.61, 1.53)	0.87
Maternal knowledge that TANF and Medicaid eligibility criteria differ						
Yes	Ref.	–	Ref.	–	Ref.	–
No	**1.47(1.02, 2.13)**	**0.038**	1.33(0.87, 2.05)	0.18	1.27(0.78, 2.07)	0.34
Maternal self-reported health^*^						
Total < 80 (poor health)	Ref.	–	Ref.	–	Ref.	–
Total ≥ 80 (good health)	0.95(0.67, 1.34)	0.76	0.94(0.63, 1.40)	0.76	0.74(0.61, 1.44)	0.78
Prenatal care, self-reported^*^						
All/Most of the time	Ref.	–	Ref.	–	Ref.	–
Some or none of the time	0.81(0.46, 1.41)	0.45	0.79(0.44, 1.44)	0.44	0.70(0.33, 1.50)	0.36
Maternal social support^*^						
Low	Ref.	–	Ref.	–	Ref.	–
Medium	0.74(0.46, 1.18)	0.20	0.85(0.51, 1.41)	0.53	1.04(0.52, 2.08)	0.92
High	1.06(0.69, 1.62)	0.80	1.35(0.83, 2.20)	0.23	**1.91(1.03, 3.52)**	**0.04**
Not collected	**0.37(0.21, 0.65)**	**0.0006**	**0.33(0.16, 0.68)**	**0.003**	**0.14(0.04, 0.51)**	**0.003**
Household income^*^						
< $1000/month	Ref.	–	Ref.	–	Ref.	–
$1000 or more /month	1.05(0.74, 1.50)	0.78	1.00(0.68, 1.46)	0.99	1.39(0.90, 2.16)	0.14
Maternal employment status^*^						
Student	Ref.	–	Ref.	–	Ref.	–
Employed	1.33(0.89, 1.98)	0.16	1.42(0.92, 2.20)	0.12	1.18(0.71, 1.96)	0.52
Unemployed	0.95(0.62, 1.47)	0.83	1.06(0.66, 1.70)	0.81	0.85(0.50, 1.96)	0.55
Other children in household						
None	Ref.	–	Ref.	–	Ref.	–
One	0.76(0.51, 1.14)	0.18	0.90(0.58, 1.40)	0.64	0.64(0.38, 1.09)	0.098
Two or more	0.72(0.50, 1.05)	0.089	0.86(0.57, 1.30)	0.47	0.88(0.55, 1.40)	0.59
Family housing situation^*^						
Lives in own housing	Ref.	–	Ref.	–	Ref.	–
Rents or lives with relatives/friends	**1.42(1.01, 1.99)**	**0.04**	1.34(0.93, 1.93)	0.11	1.43(0.94, 2.17)	0.09
Travel time to Medicaid office^*^						
≤ 30 min	Ref.	–	Ref.	–	Ref.	–
> 30 min	1.01(0.70, 1.46)	0.97	1.16(0.79, 1.70)	0.44	1.17(0.73, 1.86)	0.50

Note: Maternal disenrollment was defined as having any gap in Medicaid coverage. Maternal health literacy was assessed using the S-TOFHLA and categorized as inadequate, marginal, or adequate per published technical guidance (Nurss JR, Parker R, Willams M, Baker D. TOFHLA: Test of Functional Health Literacy in Adults. Second ed. Snow Camp, NC: Peppercorn Books & Press; 2001). Maternal instrumental and relational social support was assessed using the Maternal Social Support Index and categorized low, medium, or high using tertiles per published technical guidance (Pascoe JM, Ialongo NS, Horn WF, Reinhart MA, Perradatto D. The reliability and validity of the maternal social support index. *Fam. Med*. Jul-Aug 1988;20(4):271–27)

^*^Results from 10 imputed datasets

Entries in boldface have *p*-values less than 0.05

Maternal disenrollment and mother-child cash assistance receipt were also significantly associated with time to child’s first Medicaid disenrollment (Table 3). Maternal disenrollment was associated with a more than 5 times increased rate of child disenrollment. Children whose families were renting or living with friends/relatives had larger rate of disenrollment than those living in their own housing. Children living in households without any other children also had increased rate of Medicaid disenrollment.

**Table 3:** Hazard ratios for predictors of child’s time to first Medicaid disenrollment based on single predictor variable Cox proportional hazard models

Variable	Time to First Disenrollment	
	Hazard Ratio (95% CI)	P-value
Maternal disenrollment		
Under Medicaid coverage	Ref.	–
Disenrolled from Medicaid (any gap)	**5.48 (4.02, 7.46)**	**< 0.001**
Child cash assistance status		
No	Ref.	–
Yes	**0.31 (0.23, 0.42)**	**< 0.001**
Mother cash assistance status		
No	Ref.	–
Yes	0.86 (0.66, 1.13)	0.29
Combined cash assistance status		
Neither had cash assistance	Ref.	–
Only mother had cash assistance	1.32 (0.95, 1.84)	0.10
Only child had cash assistance	**0.31 (0.20, 0.48)**	**< 0.001**
Both had cash assistance	**0.36 (0.24, 0.52)**	**< 0.001**
Maternal age		
	0.98 (0.96, 1.01)	0.19
Maternal race		
African American	Ref.	–
Other	1.33 (0.96, 1.84)	0.09
Marital status		
Single/widowed/divorced	Ref.	–
Married	1.28 (0.88, 1.86)	0.20
Maternal health literacy^*^		
Inadequate	Ref.	–
Marginal	0.74 (0.48, 1.13)	0.16
Adequate	0.66 (0.38, 1.14)	0.13
Maternal education		
Less than High School	Ref.	–
High School	0.97 (0.67, 1.40)	0.88
More than High School	0.90 (0.63, 1.21)	0.48
Maternal knowledge that TANF and Medicaid eligibility criteria differ		
Yes	Ref.	–
No	1.35 (0.98, 1.85)	0.07
Maternal self-reported health^*^		
Total < 80 (poor health)	Ref.	–
Total ≥ 80 (good health)	0.85 (0.64, 1.13)	0.26
Prenatal care^*^		
All/Most of the time	Ref.	–
Some or none of the time	1.04 (0.62, 1.72)	0.89
Maternal social support^*^		
Low	Ref.	–
Medium	0.78 (0.51, 1.21)	0.27
High	1.10 (0.78, 1.57)	0.58
Not collected	1.47 (0.86, 2.52)	0.16
Household income^*^		
< $1000/month	Ref.	–
$1000 or more /month	0.91 (0.68, 1.22)	0.53
Maternal employment status^*^		
Student	Ref.	–
Employed	0.91 (0.65, 1.29)	0.92
Unemployed	0.80 (0.55, 1.18)	0.80
Other children in household		
None	Ref.	–
One	**0.59 (0.42, 0.82)**	**0.002**
Two or more	**0.64 (0.47, 0.87)**	**0.005**
Family housing situation^*^		
Lives in own housing	Ref.	–
Rents or lives with relatives/friends	**1.64 (1.26, 2.14)**	**0.0003**
Travel time to Medicaid office^*^		
≤ 30 min	Ref.	–
> 30 min	1.01 (0.74, 1.37)	0.96

Note: Maternal disenrollment was defined as having any gap in Medicaid coverage. Maternal health literacy was assessed using the S-TOFHLA and categorized as inadequate, marginal, or adequate per published technical guidance (Nurss JR, Parker R, Willams M, Baker D. TOFHLA: Test of Functional Health Literacy in Adults. Second ed. Snow Camp, NC: Peppercorn Books & Press; 2001). Maternal instrumental and relational social support was assessed using the Maternal Social Support Index and categorized low, medium, or high using tertiles per published technical guidance (Pascoe JM, Ialongo NS, Horn WF, Reinhart MA, Perradatto D. The reliability and validity of the maternal social support index. *Fam. Med*. Jul-Aug 1988;20(4):271–27)

^*^Results from 10 imputed datasets

Entries in boldface have *p*-values less than 0.05

The main findings from the single variable analyses persisted in the multivariable analyses with a few notable differences. Using the best subsets approach, maternal disenrollment and mother-child dyad cash assistance status, but not maternal health literacy, were included in the final model (Table 4). Though not significant in single variable results, monthly household income was included in the final best subsets models for any gap and > 14 day gaps because household income is an explicit criterion for Medicaid eligibility. Controlling for relevant covariates, children of mothers who disenrolled from Medicaid had 10 times greater odds of disenrollment than children with insured mothers at all three thresholds used to define coverage gaps. Particular combinations of maternal-child cash assistance receipt remained protective for any gap and gaps > 14 days, but not for gaps > 60 days. Consistent with single variable results, maternal knowledge that TANF and Medicaideligibility criteria differ and monthly household income remained associated with child disenrollment.

**Table 4:** Odds ratios for predictors of child Medicaid disenrollment from best-fitting GEE models

Variable	Any gap			> 14-day gap			> 60-day gap		
	Odds Ratio	95% confidence interval	*P*-value	Odds Ratio	95% confidence interval	*P*-value	Odds Ratio	95% confidence interval	*P*-value
Maternal disenrollment									
Under Medicaid coverage	Ref.	–	–	Ref.	–	–	Ref.	–	–
Disenrolled from Medicaid (any gap)	**12.60**	**(8.11, 19.58)**	**< 0.001**	**11.78**	**(7.38, 18.82)**	**< 0.001**	**16.75**	**(9.67, 29.02)**	**< 0.001**
Maternal knowledge that Medicaid and TANF eligibility criteria differ									
Yes	Ref.	–	–	Ref.	–	–	–	–	–
No	**2.01**	**(1.21, 3.35)**	**0.007**	**1.81**	**(1.05, 3.13)**	**0.03**	–	–	–
Combined cash assistance status									
Neither had cash assistance	Ref.	–	–	Ref.	–	–	Ref.	–	–
Only mother had cash assistance	1.85	(0.99,3.41)	0.05	**2.11**	**(1.11, 3.99)**	**0.02**	2.15	(0.95, 4.86)	0.07
Only child had cash assistance	**0.38**	**(0.19, 0.76)**	**0.006**	**0.48**	**(0.23, 0.98)**	**0.04**	0.50	(0.22, 1.14)	0.10
Both had cash assistance	**0.48**	**(0.29, 0.82)**	**0.007**	0.59	(0.34, 1.04)	0.07	0.78	(0.41, 1.46)	0.43
Household income^*^									
< $1000/month	Ref.	–	–	Ref.	–	–	–	–	–
$1000 or more /month	**0.59**	**(0.36, 0.96)**	**0.03**	**0.57**	**(0.34, 0.94)**	**0.03**	–	–	–
Family housing situation^*^									
Lives in own housing	–	–	–	–	–	–	Ref.		–
Rents or lives with relatives/friends	–	–	–	–	–	–	**2.08**	**(1.21, 3.56)**	**0.008**

Note: Maternal disenrollment was defined as having any gap in Medicaid coverage. All models were based on 604 dyads. The best fitting model was selected based on the QIC and the QICu. All final models were checked to ensure that adding another variable did not significantly change the QIC or the QICu

^*^Results from 10 imputed datasets

Entries in boldface have *p*-values less than 0.05

In the adjusted models analyzing time to the child’s first Medicaid disenrollment, maternal disenrollment was associated with a more than 4 times increased rate of child disenrollment. Consistent with our findings using odds ratios, children receiving cash assistance and those whose families had higher household income demonstrated lower rates of disenrollment (Table 5).

**Table 5:** Hazard ratio for child’s time to first disenrollment

Variable	Time to first disenrollment		
	Hazard Ratio	95% confidence interval	P-value
Maternal disenrollment			
Under Medicaid coverage	Ref.	–	–
Disenrolled from Medicaid (any gap)	**4.80**	**(3.48, 6.61)**	**< 0.001**
Household income*			
< $1000/month	Ref.	–	–
$1000 or more /month	**0.62**	**(0.46, 0.83)**	**0.0016**
Combined cash assistance status			
Neither had cash assistance	Ref.	–	–
Only mother had cash assistance	1.28	(0.92, 1.78)	0.15
Only child had cash assistance	**0.38**	**(0.24, 0.59)**	**< 0.001**
Both had cash assistance	**0.52**	**(0.35, 0.78)**	**0.001**

Note: Maternal disenrollment was defined as having any gap in Medicaid coverage

^*^Results from 10 imputed datasets

Entries in boldface have *p*-values less than 0.05

## Discussion

In this study population, maternal Medicaid enrollment status was significantly and strongly associated with child Medicaid enrollment status. This association between maternal disenrollment and child disenrollment remained strong and significant for gaps of any length and after adjusting for relevant covariates. Consistent with our hypotheses and Aday and Anderson’s framework [8], maternal and child cash assistance receipt and maternal knowledge about differences in eligibility criteria for Medicaid and TANF were significantly associated with child Medicaid enrollment status. As expected, maternal disenrollment, household income, and cash assistance receipt are associated with time to child’s first disenrollment. Our findings are consistent with other studies [11, 15] that together underscore the importance of supporting family coverage and continued outreach efforts to potential eligible populations in order to improve child Medicaid retention.

We found that a greater proportion of mothers in the cohort experienced disenrollment than children. The higher rate of unstable Medicaid coverage for mothers may be related to a more burdensome application process and/or differences in income eligibility thresholds for adults than for children. During 2005–2006, there were only 27 states that had family-friendly applications where parents could complete a single application for their child and themselves [33]. While only six states required an asset test for child Medicaid applications, 30 states required asset tests for parent Medicaid applications [33]. In the wake of the 2010 Affordable Care Act (ACA) implementation, states have focused on further streamlining the Medicaid application and renewal processes by leveraging technology and using a single application for the entire family such that the children’s uninsured rate reached a historic low of less than 5% [34]. Our findings indicate that repealing the ACA Medicaid expansion is likely to have adverse impact on child Medicaid enrollment. It is unclear whether policy makers will continue to support ACA expansions and streamlining Medicaid application processes in the long-term.

Mother-child cash assistance receipt and child cash assistance receipt had strong protective effects against child disenrollment. At the same time, about 20% of the mothers in this study did not know that Medicaid and TANF had separate eligibility processes. The TANF enrollment process is as complicated, if not more so, as the Medicaid enrollment process [11, 13]. One plausible explanation for our finding is that parents who were able to navigate the cash assistance application process were also more likely to know how to navigate the Medicaid application process, thus lowering the likelihood of the child’s disenrollment from Medicaid. Since the ACA was implemented, states have improved outreach efforts to assist eligible parents and children to enroll in Medicaid. Most states now offer web-based accounts to manage Medicaid coverage after enrollment and more than half have a portal that enables consumer assisters to submit applications on behalf of individuals that they help [35]. Effective outreach and enrollment efforts will be needed to continue to reach eligible families, both old and new, to facilitate enrollment in expanded Medicaid programs.

We also found that the set of predictors significantly associated with child Medicaid disenrollment changed when the threshold for defining gaps lengthened. Specifically, using > 60 days as the threshold for defining gaps resulted in family housing situation becoming a significant predictor whereas household monthly income and maternal knowledge that Medicaid and TANF have different eligibility criteria did not remain significant. This change in predictors suggests that families whose children had longer gaps face different barriers to Medicaid renewal than families whose children had shorter gaps. These findings are consistent with results from other states [4, 16] and suggest different outreach and assistance efforts – such as targeted assistance to maintain coverage for families in unstable housing--- are needed when trying to reach families of children with different lengths of coverage gaps.

There are some limitations to this study. First, child Medicaid enrollment patterns were only observed for its first 24 months of life. As children grow older and family characteristics change, the relationship between child and maternal Medicaid enrollment patterns is also likely to weaken. Notably, in this study, we do not assess the types of disenrollment (e.g., increased household income, termination of emergency Medicaid, etc.) and maternal disenrollment is not a random event. However, from the perspective of patients and providers, nearly one-quarter of low-income adults still experience ‘churning,’ (i.e. moving between and out of health plans) in the post-ACA era with adverse consequences including disrupted care and medication adherence, increased emergency department use, and worsening self-reported quality of care [36–38]. Second, this study cohort is primarily comprised of African-American families living in an urban area. Further studies among diverse populations are needed to assess generalizability of these findings. Third, we assessed maternal health literacy using only the S-TOFHLA and did not find a significant association between health literacy and child Medicaid disenrollment. A recent review of 19 health literacy indices concluded that none of the currently available health literacy measures fully assesses a person’s ability to obtain, process, and understand health information, however the TOFHLA demonstrates the strongest psychometric properties of all the instruments examined [39].

## Conclusions

We found that maternal Medicaid disenrollment is associated with a more than 10 times increased odds of child Medicaid disenrollment, regardless of the duration of the gap. Children who experienced shorter gaps in coverage faced some different barriers than children who experienced longer gaps in coverage. With ACA currently in effect, many more new families are eligible for publicly funded health insurance, in addition to those eligible families who were not previously enrolled. To ensure all eligible families can take advantage of these coverage opportunities, policymakers need to invest in effective and appropriate outreach strategies and provide family-friendly application processes to reduce enrollment barriers.

## Appendix 2

**Table 6:** Characteristics of children with and without Medicaid administrative eligibility data

	Have Medicaid Data (*N* = 604) N(%)	No Medicaid Data (*N* = 140) N(%)	*P*-value
Mean Maternal Age (SD)			
	23.2 (5.2)	24.8 (5.8)	0.0035
Maternal Race			
African American	569 (94)	137 (98)	0.03
Other	35 (6)	3 (2)	
Other children			
None	228(38)	43(31)	0.29
One	157(26)	41(29)	
Two or more	219(36)	56(40)	
Missing	0	2	
Education			
Less than high school	198(33)	45(32)	0.98
High school	147(24)	35(25)	
More than high school	259(43)	60(43)	
Health Literacy			
Inadequate	54(9)	15(11)	0.33
Marginal	77(13)	12(9)	
Adequate	449(77)	109(80)	
Missing	24	4	
Prenatal Care			
All/Most of the time	552(92)	128(91)	0.96
Some/None of the time	51(8)	12(9)	
Missing	1	0	
Income			
< $1000/month	424(76)	102(76)	0.86
$1000 or more/month	132(24)	33(24)	
Missing	48	5	
Marital Status			
Single/Divorced/Widowed	533 (88)	113(82)	0.05
Married	71 (12)	25(18)	
Missing	0	2	
Country			
US Born	573(95)	112(80)	< 0.0001
Non-US Born	31(5)	28(20)	
Maternal Social Support Index			
Low	151(34)	32(36)	0.83
Medium	148(33)	31(35)	
High	144(33)	26(29)	
Not Applicable	126	39	
Missing	35	12	

Note: P-value was for the exact χ^2^ test of association for categorical variables. T-test was used for maternal age

## Appendix 3 Imputation Methods

As with typical pattern of missingness of responses in surveys, many subjects failed to complete or respond properly to one or more item. The fraction of missing survey data ranged from 0.17% to 16.06% per item, with variables for maternal employment and knowledge that Medicaid and TANF eligibility criteria differ having more than 10% missing. For each of the chained equations, we selected those other variables that in theory would predict the unobserved values. This method requires that each variable with a missing value be specified in a regression equation with the proper form (e.g., nominal, binary, etc.) for the missing values. Continuous variables were imputed using a linear regression model, binary variables with missing values were imputed using a logistic regression, while those with nominal values used multinomial logit regression. Several variables were clearly ordered (e.g., education, income category) and were imputed using ordinal logistic regression. The chained equation methods imputes one value at a time, and then with the value filled in, proceeded to the next missing variable and its equation, which then assumed that the imputed value is known. At each imputation, the value chosen is taken from the posterior predictive distribution of from the regression, and unlike simple mean or regression-based imputation, this method adequately accounts for the additional variance arising when values must be filled in. The chain of equations is then repeated in 10 cycles to achieve better convergence. The entire process of 10 cycles is then repeated 10 times to obtain 10 sets of imputed values. For each of the chained equations, we selected variables that in theory would predict the unobserved values. In addition, as is proper for imputation, we included maternal age, child gender, and whether the child disenrolled at any time as auxiliary variables. This imputation permits the proper estimation of variance by combining two variance components: the average of the within imputation variances and the across imputation variance. ^32^ All reported results are from completed datasets using the imputation procedures previously described and analyzed in SAS® v9.1.

## Abbreviations

ACA: Affordable Care Act
AIC: Akaike information criterion
GEE: Generalized estimating equations
HIP: Health Insurance Improvement Project
MSSI: Maternal Social Support Index
OR: Odds ratio
S-TOFHLA: Short-Test of Functional Health Literacy in Adults
TANF: Temporary Assistance to Needy Families
